# The accuracy and effectiveness of automatic pedicle screw trajectory planning based on computer tomography values: an in vitro osteoporosis model study

**DOI:** 10.1186/s12891-022-05101-6

**Published:** 2022-02-21

**Authors:** Jia Bin Liu, Rui Zuo, Wen Jie Zheng, Chang Qing Li, Chao Zhang, Yue Zhou

**Affiliations:** Department of Orthopaedics, Xinqiao Hospital, Amy Medical University (Third Military Medical University), Chongqing, 400037 People’s Republic of China

**Keywords:** Osteoporosis, Pedicle screw placement, Software automatic planning, Biomechanical research, In vitro model test

## Abstract

**Background:**

Pedicle screw placement in patients with osteoporosis is a serious clinical challenge. The bone mineral density (BMD) of the screw trajectory has been positively correlated with the screw pull-out force, while the computer tomography (CT) value has been linearly correlated with the BMD. The purpose of this study was to establish an in vitro osteoporosis model and verify the accuracy and effectiveness of automated pedicle screw planning software based on CT values in this model.

**Methods:**

Ten vertebrae (L1-L5) of normal adult pigs were randomly divided into decalcification and control groups. In the decalcification group, the vertebral bodies were decalcified with Ethylenediaminetetraacetic acid (EDTA) to construct an in vitro osteoporosis model. In the decalcification group, automatic planning (AP) and conventional manual planning (MP) were used to plan the pedicle screw trajectory on the left and right sides of the pedicle, respectively, and MP was used on both sides of the control group. CT values of trajectories obtained by the two methods were measured and compared. Then, 3D-printed guide plates were designed to assist pedicle screw placement. Finally, the pull-out force of the trajectory obtained by the two methods was measured.

**Results:**

After decalcification, the BMD of the vertebra decreased from − 0.03 ± 1.03 to − 3.03 ± 0.29 (*P* < 0.05). In the decalcification group, the MP trajectory CT value was 2167.28 ± 65.62 Hu, the AP trajectory CT value was 2723.96 ± 165.83 Hu, and the MP trajectory CT value in the control group was 2242.94 ± 25.80 Hu (*P* < 0.05). In the decalcified vertebrae, the screw pull-out force of the MP group was 48.6% lower than that of the control group (*P* < 0.05). The pull-out force of the AP trajectory was 44.7% higher than that of the MP trajectory (*P* < 0.05) and reached 97.4% of the MP trajectory in the control group (*P* > 0.05).

**Conclusion:**

Automatic planning of the pedicle screw trajectory based on the CT value can obtain a higher screw pull-out force, which is a valuable new method of pedicle screw placement in osteoporotic vertebre.

**Supplementary Information:**

The online version contains supplementary material available at 10.1186/s12891-022-05101-6.

## Background

Osteoporosis is a metabolic bone disease characterized by low bone mass. It is a common disease in elderly people, especially in postmenopausal women, and can cause changes in the biomechanical properties of the vertebrae [1, 2]. In conditions requiring pedicle screw placement, the intensity of pedicle screw fixation may be reduced, loosened, and pulled out, which eventually leads to failure of the operation [3, 4]. Many studies have been conducted to increase the local tissue density of the vertebrae by increasing the screw length, diameter, and thread structure or by injecting bone cement to increase the pull-out force of screws [5, 6]. However, these methods all have shortcomings. Cement-augmented screws carry the risk of cement leakage which the most serious complications are spinal cord compression and pulmonary embolism [7]. The increase in screw diameter may be accompanied by the occurrence of pedicle microfractures, leading to a decrease in the screw pull-out force [8, 9].

The placement position of the pedicle screw is a crucial factor affecting the pull-out force of the screw, and its accuracy is related not only to malposition but also to the biomechanical function of the pedicle screw [10]. Previous studies have demonstrated a linear relationship between the CT value and BMD and an exponential relationship between BMD and Young’s modulus of bone tissue [11–15]. Therefore, placing the pedicle screw in a position where the CT value of the screw trajectory is as large as possible may be another method of pedicle screw placement for osteoporosis.

The application of navigation-guidance technology based on CT images can provide a holistic anatomical image of the surgical area through a single scan. It could prevent repeated fluoroscopy during the operation, and locate and track the relative position of surgical instruments and anatomical structures in real-time, thus avoiding pedicle perforation and effectively reducing the occurrence of nerve and vascular complications caused by pedicles [16]. However, these assistive techniques require preoperative or intraoperative analysis of the patient’s CT images and manual planning of the entry point and trajectory of pedicle screws by the surgeon, which is a complicated and time-consuming process. Moreover, CT images alone are not enough for the surgeon to consider whether the bone mass at the screw trajectory provides adequate fixation of the pedicle screw, especially in patients with osteoporosis. With the development of computer technology, more powerful computers and better algorithms have been developed. These developments allowed automated planning software could find a trajectory that meets the highest CT value and does not violate the placement standard. Several auto planning methods have been designed [17–20], but the studies were not planned in a way that specifically targeted osteoporosis patients, and most studies have not conducted in vitro experiments to verify their accuracy and screw pull-out force.

To address these problems, this study used an automatic planning software to analyze CT data on an osteoporosis vertebra model to search trajectories of the highest CT value. Trajectories with non-perforating pedicles and non-piercing vertebrae were found to obtain a better effect of screw fixation. The in vitro osteoporosis model was used to verify its accuracy and effectiveness. Surgeons who find pedicle screw placement difficult in patients with osteoporosis may be interested in this research, which may provide them with a potentially effective solution to avoid more implants.

## Methods

### Establishment of osteoporosis model in vitro

Ten lumbar vertebrae were taken from mature pigs (L1-L5, weighing 120–130 kg). All pigs were healthy, and none had been exposed to any factors that might affect BMD. All vertebrae were stripped of surrounding muscle tissue, ligaments, and periosteum. Parts of the spinous and transverse processes were excised and embalmed with 10% paraformaldehyde for 48 h. Micro-CT (μCT) was used for detection. Five of the 10 vertebrae were used as a decalcification group, and the other 5 were used as a control group. Five vertebrae were randomly selected from all specimens using Ethylenediaminetetraacetic acid (EDTA) decalcified fluid for decalcification. A 3 mm *10 mm hole was drilled in the upper and lower endplates of the vertebral body. During the decalcification process, a stepper motor was connected to these holes through a silicone tube, and EDTA was pumped at a rate of 1 ml/min. This procedure allowed the cancellous bone and cortical bone of the vertebrae to be decalcified simultaneously. Decalcification was performed for 2–3 weeks. The EDTA solution was replaced weekly, and the vertebrae were scanned weekly with μCT until the osteoporosis criteria were met.

The BMD level was expressed by T value, T value = (measured value – average normal BMD)/standard deviation of normal BMD. Due to the linear relationship between the CT value and BMD, the CT value of normal vertebrae could be used as a substitute value for normal BMD. The formula was rewritten as T’ value = (measured CT value – average value of normal vertebral CT value)/standard deviation of normal vertebral CT value. Osteoporosis was defined when the T’ value ≤ − 2.5.

### Trajectory planning of pedicle screw placement

Software automatic planning and manual planning were used for pedicle screw trajectory planning, respectively. Software automatic planning was used for the left pedicle, and manual planning was used for the right pedicle. All trajectories were required not to pierce the pedicle and anterior vertebral margin. To ensure the uniformity of biomechanical testing, the specifications of all planned trajectories were unified as 6 mm * 30 mm.

#### Automatic planning

Finav (Version 1.0, Chongqing Bosscom Technology Co., Ltd., Chongqing, China) was used for automatic planning based on CT values. The surgeon only needs to manually adjust the area of interest to mark the target vertebral body and the narrowest part of the pedicle and specify the screw diameter and length according to the recommended screw diameter and length calculated by the software. Then, the software can automatically calculate and plan the screw trajectory with the largest CT value [21]. The software used an optimization algorithm to address the optimization problem of maximizing the mean CT value of the pedicle screw trajectory under a set of relevant constraints. The software was in the developing and testing stage, and this study was designed to study its accuracy and effectiveness. After its function was verified, the software will be added as a functional module in the optical and magnetic integrated surgical navigation system of Chongqing Bosscom Technology Co., Ltd.

#### Conventional manual planning

The artificially planned trajectory passed through the midpoint of the minimum section of the pedicle. The direction was parallel to the endplate on the vertebral body with an angle position the screw on the central axis of the pedicle. The same senior spine surgeon manually planned all trajectories.

####  Measurement of position and CT values of different planned trajectories 

**Fig. 1:**
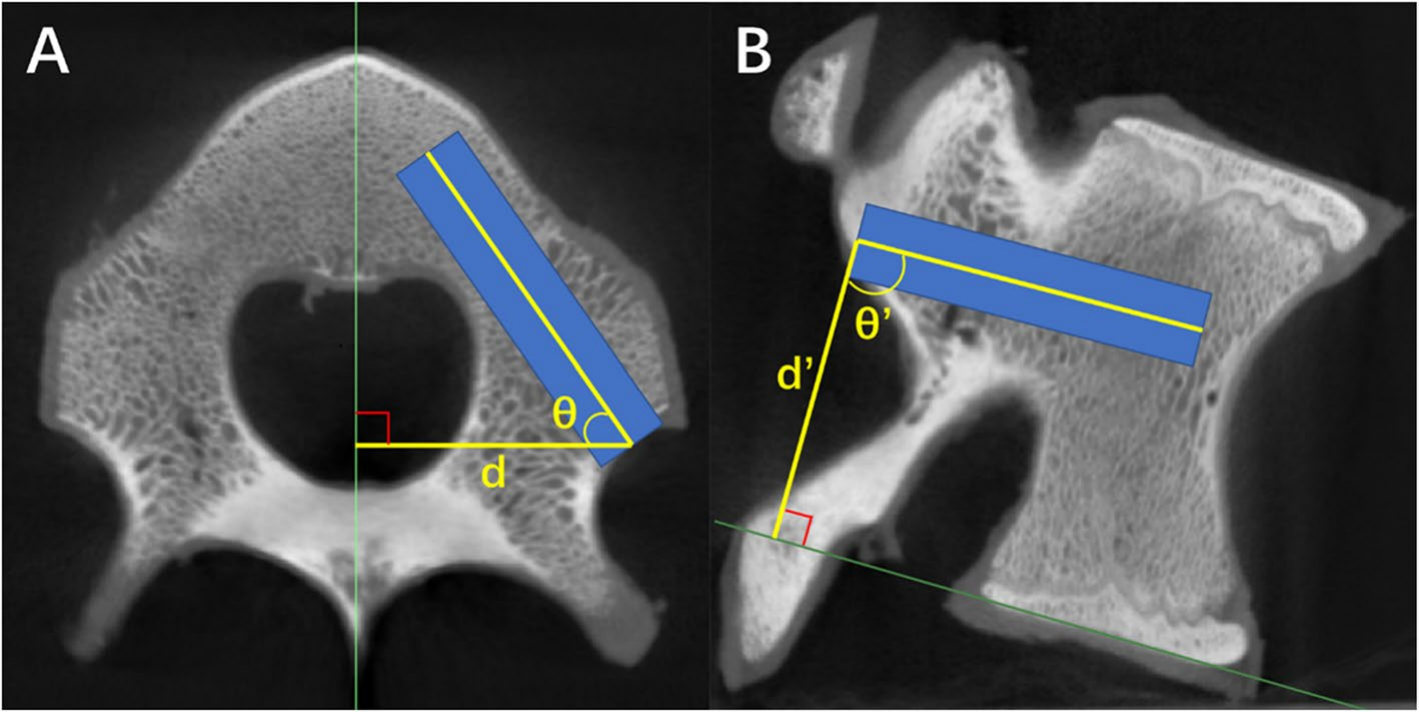
Measurement of pedicle screw planning trajectory. **A** Axial view of the vertebra. Draw a guideline (green straight line) through the midline of the vertebra. Measure the vertical distance d from the entry point to the midline and the angle θ between the long axis of the screw and d. **B** Sagittal view of the vertebra. Draw a guideline (green straight line) along the lower endplate of the vertebral body. Measure the vertical distance d’ between the entry point of the screw and the guideline and the angle θ’ between the long axis of the screw and d’

The position of the screw trajectory was measured on axial and sagittal CT views [22]. Draw guides (green) along the midline of the axial view (bisect the vertebral body, spinal canal, and spinous process) and the lower endplate of the sagittal view is shown, where d represents the distance from the insertion point to the sagittal midline of the vertebral body and θ represents the angle of the screw in the axial position. D′ represents the sagittal distance from the entry point to the lower endplate, and θ’ represents the sagittal angle of the screw. (90°-θ) is the abduction Angle of the screw. (θ ‘-90°) is the craniocaudal inclination of the screw, with a positive value representing caudal inclination and a negative value representing cephalic inclination (Fig. 1).

Mimics software (Version 21, Materialise, Belgium) was used to analyze the μCT data of the osteoporosis model, the trajectory of the automatic planning and manual planning were analyzed, and the CT values of the trajectory obtained by different planning methods were measured.

### Placement of pedicle screws

#### Design and manufacture of 3D printed guide plate

In this study, 3D printed guide plates were used to realize planned trajectories. Mimics and 3-Matic (Version 13, Materialise, Belgium) software were used to import CT data, and 3D image reconstruction was conducted. The 3D printing guide plates were designed according to the screw entry points and stop point coordinates obtained by automatic and manual planning. The automatic planning trajectories were on the left side, and manual planning trajectories were on the right side. The designed 3D printing guide plates were imported into the 3D printer software, the printing parameters were adjusted, and the plates were printed.

#### Pedicle screw insertion

Pedicle screw placement was assisted by 3D-printed guide plates. A 2.5 mm Kirschner wire was used to turn the hole through the guide head of the 3D-printed guide plate to obtain the screw trajectory, and a pedicle screw was inserted along the trajectory (6 mm*30 mm, Sanyou, Shanghai, China). The same screw specifications were used to obtain comparable results in subsequent pull-out tests.

#### Measurement of pedicle screw placement error

After placement, X-rays, and CT were used to verify the position of the pedicle screw to ensure that the pedicle screw was completely located in the pedicle without penetrating the pedicle and the vertebral cortex. The screw placement error was measured by preoperative and postoperative CT images. Similar to 2.3, d1 and θ1 represent the actual screw position as measured by postoperative CT. The displacement error is (d1-d); the angle error is (θ1-θ).

### Screw pull-out test

Each vertebra was fixed so that the long axis of the pedicle screw was coaxial with the direction of tension (ElectroPuls E10000 All-Electric Dynamic Test Instrument, Instron Industrial Products, Grove City, USA). The axial pull-out test was performed at a speed of 5 mm/min, and the force applied to the screw was recorded in displacement increments of 0.05 mm until the screw was completely removed. The maximum load encountered during the test was defined as the peak load (pull-out force) at the time of failure. The test sequence of the left and right pedicles was alternated with each specimen to eliminate the effect of the test sequence.

### Statistical analysis

SPSS (version 23.0, SPSS Inc., Chicago, IL, USA) was used for statistical analysis, and the data were expressed as the mean ± standard deviation. The normality of the two sets of continuous variables was assessed by the Shapiro-Wilk test (W test), and the F-test was used to determine whether the overall variance of the two sets of continuous variables was homogeneous. If the data were normally distributed and the variance was homogeneous, A paired sample T-test was used for pairwise comparisons of decalcification group data, and an independent sample T-test was used for intergroup comparisons. *P* < 0.05 indicated a significant difference.

## Results

### Results for the establishment of the osteoporosis model

The CT values of all vertebral bodies were 2767.87 ± 26.15 Hu. After 2 weeks of EDTA decalcification, the CT value of the decalcification group decreased from 2767.15 ± 27.08 Hu to 2688.8 ± 7.67 Hu (*P* < 0.05), and the T’ value decreased from − 0.03 ± 1.03 to − 3.03 ± 0.29 (*P* < 0.05). All the vertebral bodies in the decalcification group met the standard of osteoporosis (T’ < -2.5) (Table 1). See Supplementary Table 1, Additional file 1 for detailed data.

**Table 1:** Measurement of mean CT value of vertebrae

	CT value (Hu)	T’ value
Decalcification group (*n* = 5)		
Before decalcification ^a^	2767.15 ± 27.08^b^	−0.03 ± 1.03^b^
After decalcification ^b^	2688.80 ± 7.67^acd^	− 3.03 ± 0.29^acd^
Control group (*n* = 5)^c^	2768.59 ± 28.35^b^	0.03 ± 1.08^b^
All samples (*n* = 10)^d^	2767.87 ± 26.15^b^	0.00 ± 1.00^b^

^a^represents the *P* < 0.05 compared with the decalcification group before decalcification, ^b^represents the *P* < 0.05 compared with the decalcification group after decalcification, ^c^represents the *P* < 0.05 compared with the control group, and ^d^represents the *P* < 0.05 compared with all samples without decalcification

### Measurements of the position and CT value of the pedicle screw planning trajectory

The automatically planned screw trajectory was imported into the reconstructed CT image, and whether the screw perforated the lateral wall of the pedicle and the anterior edge of the vertebral body was evaluated from the axial view. None of the screws, whether automatic planning or manual planning, resulted in pedicle perforation or vertebral anterior edge perforation. The axial angle, coronal distance, and coronal angle of the screw entry point in automatic planning were more significant than those in manual planning (*p* < 0.05), while the axial distance was less than that in manual planning (*p* < 0.05) (Table 2). The automatic planned screw entry point is inward and upward, and the trajectory has a larger tail inclination and a smaller abduction angle. See Supplementary Table 2, Additional file 1 for detailed data.

**Table 2:** Position measurement of planned trajectories

	Automatic planning	Manual planning	*P*-Value
Axial distance (mm)	19.47 ± 1.95	20.70 ± 2.21	0.000
Axis Angle (°)	60.43 ± 4.32	55.78 ± 4.66	0.000
Sagittal distance (mm)	22.82 ± 3.39	21.05 ± 3.06	0.000
Sagittal Angle (°)	109.19 ± 1.87	93.23 ± 1.64	0.000

In the decalcification group, the manually planned trajectory CT value was 2167.28 ± 65.62 Hu, and the automatically planned trajectory CT value was 2723.96 ± 165.83 Hu, showing a significant difference between the two groups (*P* < 0.05). It should be noted that the CT value of the manually planned trajectory in the control group was 2242.94 ± 25.8 Hu, which was higher than that in the decalcification group (*P* < 0.05) but lower than that in the automatically planned trajectory in the decalcification group (*P* < 0.05) (Table 3). See Supplementary Table 3, Additional file 1 for detailed data.

**Table 3:** CT value and screw pull-out force measurements of planned trajectories

	CT value (Hu)	Pull-out force (N)
Decalcification group (*n* = 10)		
Automatic planning (*n* = 5)^a^	2723.96 ± 165.83^bc^	1212.00 ± 143.42^b^
Manual planning (*n* = 5)^b^	2167.28 ± 65.62^ac^	837.60 ± 133.05^ac^
Control group (*n* = 10)		
Manual planning (*n* = 10)^c^	2244.76 ± 97.45 ^ab^	1244.32 ± 114.19^b^

^a^represents the *P* < 0.05 compared with the automatic planning of the decalcification group, ^b^represents the *P* < 0.05 compared with the manual planning of the decalcification group, and ^c^represents the *P* < 0.05 compared with the manual planning of the control group

### Accuracy of pedicle screw placement

**Fig. 2:**
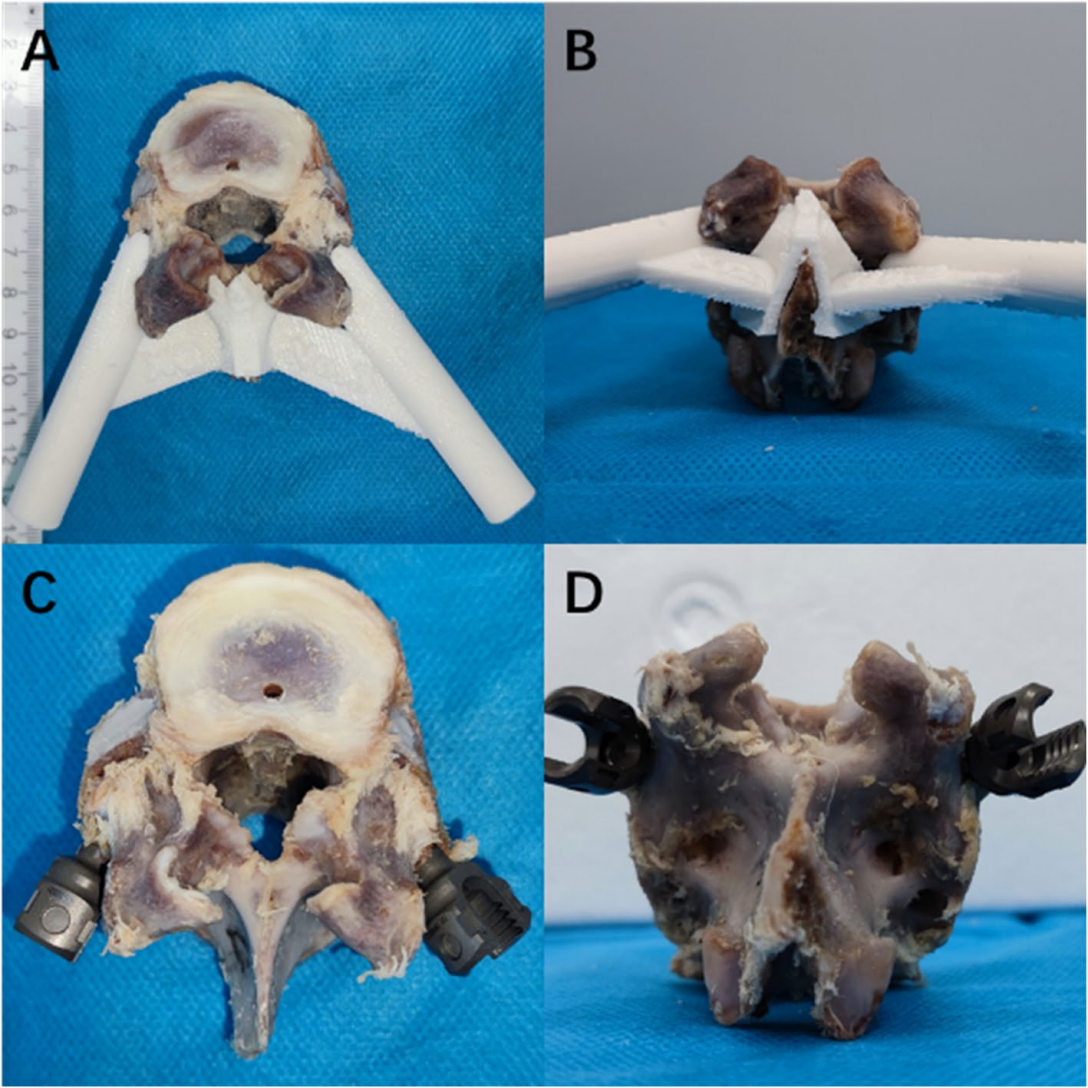
3D printed guide plate and screw implantation. **A** and (**B**) are the upper view and rear view of the 3D printed guide plate, respectively. **C** and (**D**) are the upper and rear views after screw implantation, respectively

**Fig. 3:**
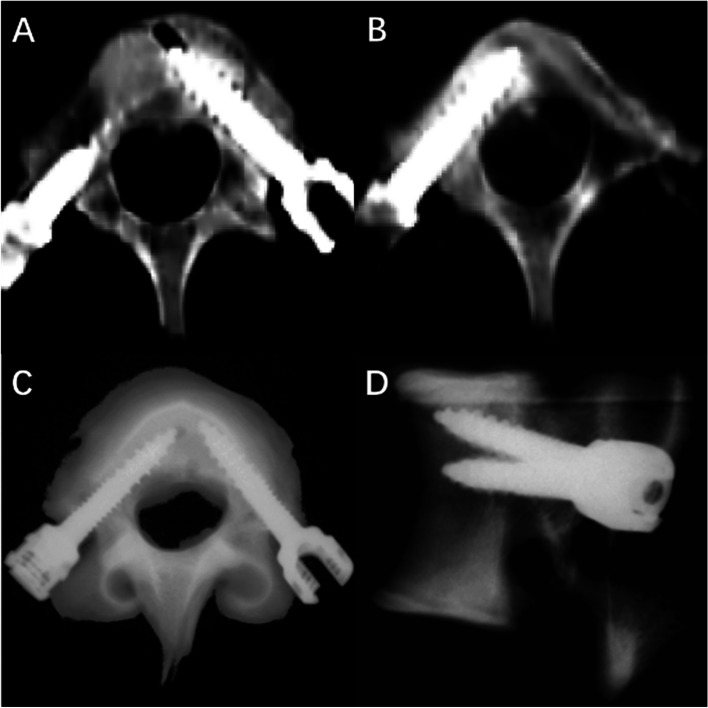
CT and X-ray detection of screw position. **A** and (**B**) are axial views of CT scans after pedicle screw implantation. **A** was the left screw view that is automatically planned, and (**B**) was the right screw view that is manually planned. **C** (**D**) was the X-ray examination after pedicle screw implantation. **C** was the bottom view of the vertebrae. The left screw of the image was the manually planned trajectory, and the right screw was the automatically planned trajectory. **D** was the lateral view of the vertebrae. The upper screw in the image was the manually planned trajectory, and the lower screw was the automatically planned trajectory. Neither screw resulted in pedicle perforation. The automatically planned trajectory has a smaller abduction angle and a larger caudal inclination angle than the manually planned trajectory

After pedicle screw placement assisted by 3D-printed guide plates, CT scans and X-ray examinations were performed to verify the accuracy of pedicle screw placement (Figs. 2 and 3). There was no significant difference between the actual and planned trajectories of all screws (*P* > 0.05) (Table 4).

**Table 4:** Measurement of screw insertion error

	Planned trajectory (*n* = 20)	Actual trajectory (*n* = 20)	*P*-value
Axial distance (mm)	20.44 ± 1.84	20.54 ± 1.85	0.252
Axis Angle (°)	58.11 ± 4.98	57.85 ± 5.46	0.395
Sagittal distance (mm)	21.11 ± 3.52	21.16 ± 3.68	0.400
Sagittal Angle (°)	101.21 ± 8.36	100.96 ± 8.65	0.065

### Comparison of pull-out force

In the decalcified osteoporotic vertebral body, the screw pull force of the manual planning group was 48.6% lower than that of the control group (*P* < 0.05). The pull-out strength of the automatically planned trajectory was 44.7% higher than that of the manually planned trajectory (*P* < 0.05) and reached 97.4% of the manually planned trajectory in the control group. There was no significant difference between the two groups (*P* > 0.05) (Table 3).

## Discussion

Pedicle screw fixation is a serious challenge in spinal surgery when confronted with osteoporosis. The in vitro osteoporosis experiment is the most direct and effective method to study the biomechanical properties of osteoporotic vertebrae. In this paper, the research was conducted by constructing in vitro models of osteoporotic pig vertebrae. In these models, the CT value and screw pull-out force of automatic trajectory planning based on the CT value and manual trajectory planning were evaluated and compared. The results showed that automatic trajectory planning was significantly better than manual trajectory planning in terms of screw pull-out force, which may suggest that automatic pedicle screw trajectory planning based on CT values is a valuable method for pedicle screw placement in osteoporosis.

The best models for studying osteoporosis in humans are from human cadavers, which are difficult to study because of their limited availability. One possible approach is to study vertebral specimens from large animals [23–25]. In this study, we used EDTA immersion and continuous pumping to demineralize pig vertebrae, which ensured that the vertebrae’s cortical bone and cancellous bone could be demineralized at the same time. This procedure significantly shortened the time needed to reach osteoporosis. Moreover, this method does not seriously damage the structure of bone trabeculae in the vertebral body and retains the biomechanical properties of the vertebral body. Lee, C. Y. et al. [26] used EDTA to decalcify the pig vertebrae and, by measuring its microstructure and mechanical properties, concluded that EDTA decalcification can help to produce a vertebral model with biomechanical properties consistent with human osteoporosis.

Pedicle screw placement in patients with osteoporosis has always been a difficult problem in spine surgery. Existing solutions include cement augmentation screw placement, cortical bone track screw placement (CBT), placement of varied materials in the screw path to increase local tissue density, increasing the screw diameter, and changing the screw thread structure. However, these methods all have their limitations. Bone cement leakage is a common complication of bone cement augmented screws, with a leakage rate of 11.6–82.4%, which can cause serious complications such as nerve injury, vascular injury, pulmonary cement embolism, cardiac embolism and anaphylactic shock [27]. In CBT, screws are inserted through the isthmus, and the screws can only be inserted into the posterior 1/3 of the vertebral body. The pull-out force is limited to the midposterior column, and the potential for correction of spinal malformation is poor. Filling the screw path with different materials, such as autologous bone, can increase the density around the screw in a certain time, but with time, the implanted material may gradually absorb or decompose, and the tissue density around the screw will gradually decrease, eventually leading to fixation failure [28]. Increasing the screw diameter and changing the screw thread structure can increase the pull-out force, but blindly increasing the screw diameter may cause pedicle fracture and decrease the pull-out force [8, 29]. It is, therefore, necessary to seek other solutions.

The development and validation of automatic pedicle screw planning systems based on preoperative or intraoperative CT are in line with the current development trend of digital and intelligent spinal surgery. Computer-assisted preoperative planning pedicle screw placement is a relatively fast process that can be performed without the automatic participation of a spine surgeon [30]. The optimization of the pedicle screw placement trajectory combined with the CT value is expected to improve the fixation effect of pedicle screws. The automated planning system reduces the time required for preoperative or intraoperative planning, optimizes the procedure of spinal navigation surgery, avoids the problem of different optimal trajectories planned by different doctors, and optimizes the fixation strength of pedicle screws. In this paper, we reported software that can plan the trajectory of the maximum pull-out force screw according to the CT value of the vertebrae and verify the safety and effectiveness of software automatic trajectory planning. Since the widely used preoperative CT and intraoperative O-arm images can only display the CT value of the tissue, this study adopted the CT value as the evaluation index to simulate the actual application scenario. Since previous studies have proven a linear relationship between the CT value and BMD, this method is feasible. In contrast to the previously reported automated planning trajectory, the automated planning trajectory used in this study did not require pedicle screws to pass through the midpoint of the pedicle, as the midpoint of the pedicle tends not to be the area with the densest bone tissue [31]. To achieve a higher extractable force of the screw, the screw needs to be as close to the cortical bone as possible without penetrating or causing the cortical bone fracture. Compared with Vijayan, R, et al. [32], this planning method does not adopt the method of establishing a model atlas for pedicle screw planning. Although building a database can greatly reduce the time required for planning, it also loses the individualized characteristics of patients. The planning method in this study was to conduct screw planning according to the actual CT reconstructed images of patients, which can avoid screw misplacement caused by failure to match the atlas due to vertebral variation.

In this study, the manual planning group adopted parallel placement technology, which inserted screw parallel to the upper endplate of the vertebral body. The sagittal angle was 3.23 ± 1.64°, while the automatic planning was 19.19 ± 1.87°, which was significantly different (*P* < 0.05), but both were within the allowable range [33]. The automatically planned trajectory is closer to the upper wall of the pedicle. Eventually, it points to the upper endplate of the vertebral body to maximize the CT value of the whole trajectory to obtain the maximum screw pull-out force. Through measurement comparison, we found that in the osteoporosis model, the trajectory CT value of the software automatic planning group was significantly higher than that of the manual planning group by 25.7% (*P* < 0.01). It should be noted that the CT values of screw tracks automatically planned in the decalcification group were 21.4% higher than those manually planned in the control group (*P* < 0.01). The use of 3D printed guide plates to place pedicle screws was exactly accurate. The displacement and angle errors of the actual screw and planned screw positions showed no significant difference (*P* > 0.05). The screws were completely located in the cortical bone, there was no perforation of the pedicle or anterior vertebral margin, and no local microfracture occurred. In vitro tests, 3D-printed guide plates are a viable alternative in scenarios where computer navigation is not available. However, in clinical application, it is impossible for surgeons to completely remove the soft tissue on the surface of the vertebrae, which makes it difficult to accurately fix the 3D-printed guide plates in the corresponding positions, resulting in the actual positions of the inserted pedicle screws significantly deviated from the planned trajectories. Therefore, the combination of automatic planning and surgical navigation systems may be the best way to use the automatic planning system in clinical practice.

Biomechanical research also proves that the automatic planning method is effective. The screw trajectory planned according to the CT value obtained a larger screw pull-out force than the traditional manually planned trajectory (*P* < 0.05). It should be noted that although the CT value of the automatically planned trajectory in the decalcification group was 21.4% higher than that of the manually planned trajectory in the control group, the screw pull-out force was only 97.4% of that in the control group. This result may suggest that the BMD of the bone tissue around the screw trajectory also influences the pull-out force of the screw, which requires further finite element analysis to explain the reason. Despite this, the automatic plan increased the pull force by 44.7% compared with the manual plan in the decalcification group, which was close to the proportion of improvement in bone cement-reinforced screws (47%) [34]. These results suggest that automatic planning based on CT values is an effective method for pedicle screw placement in osteoporotic vertebrae. This method does not require the placement of additional filler materials and avoids the serious complications caused by leakage of filler materials.

The software can export trajectory coordinates and match them with reconstructed CT images, which provides a basis for future association and matching with surgical navigation systems. In the future, combined with navigation systems or surgical robot technology, optimal trajectory planning and screw placement of the osteoporotic vertebral body can be achieved increasingly quickly, providing a new option for pedicle screw placement of the osteoporotic vertebral body.

In this study, an in vitro demineralized osteoporosis model of a porcine lumbar spine was used, and the evaluation index was only the CT value, without considering microscopic parameters such as bone trabecular structure. The structure of the pig lumbar vertebra is different from that of the human vertebra and EDTA decalcification simulates not true osteoporosis but more osteomalacia. The applicability of the pedicle screw should be verified in the patient’s CT and cadaver osteoporosis models. Specimens treated with preservatives inescapability caused changes in the material properties of specimens. Therefore, using fresh specimens to further improve the accuracy of the experimental study is necessary. In the pedicle screw pull-out experiment, we simulated direct violent pull-out, which had limited agreement with the actual pull-out situation in the human body. The pull-out force could be measured again after the cyclic fatigue test. This study specifically to age groups, requiring follow-up studies on normal vertebrae. The sample size of the test was small, and subsequent studies with larger sample sizes are needed to confirm its accuracy and effectiveness. Different designs and populations demographics to further investigate the effect of this automatic planning software are also needed.

## Conclusion

In the osteoporosis model, automatic planning of the pedicle screw trajectory based on CT value software is safe and effective without causing pedicle screw puncture. Automatic trajectory planning can obtain a higher screw pull-out force, which is 44.7% higher than manual planning and even reaches a size similar to that of normal manual planning. This method may be a valuable new method of pedicle screw placement in osteoporotic vertebral bodies.

## Supplementary Information

**Additional file 1.**

## Data Availability

The datasets used and analyzed during the current study are available from the corresponding author on reasonable request.

## Abbreviations

AP: automatic planning
BMD: bone mineral density
CT: computer tomography
EDTA: ethylenediaminetetraacetic acid
MP: manual planning
