# Supplementary material for: The accuracy and effectiveness of automatic pedicle screw trajectory planning based on computer tomography values: an in vitro osteoporosis model study
Source: BMC Musculoskelet Disord. 2022 Feb 21;23:165. doi: 10.1186/s12891-022-05101-6 (PMC8862578; doi:10.1186/s12891-022-05101-6)
Supplement: Supplementary file 1 — Additional file 1. [file 12891_2022_5101_MOESM1_ESM.docx]

Supplementary Table 1. Measurements of pig vertebrae

|  | A | B | C | D | E | F | G | H | I |
| --- | --- | --- | --- | --- | --- | --- | --- | --- | --- |
| Pig 1 |  |  |  |  |  |  |  |  |  |
| L1 | 27.88 | 19.8 | 33.99 | 9.835 | 39.75 | 8.23 | 34.9 | 39.39 | 22.92 |
| L2 | 27.57 | 18.51 | 34.64 | 9.41 | 40.685 | 8.27 | 35.905 | 41.55 | 22.84 |
| L3 | 28.13 | 18.4 | 35.86 | 15.755 | 39.28 | 7.91 | 33.83 | 49.1 | 22.65 |
| L4 | 28.07 | 18.43 | 35.72 | 16.8 | 41.71 | 8.075 | 31.2 | 50.6 | 22.23 |
| L5 | 28.84 | 18.18 | 35.14 | 17.905 | 43.77 | 8.185 | 32.39 | 52.09 | 21.62 |
| Pig 2 |  |  |  |  |  |  |  |  |  |
| L1 | 27.68 | 19.03 | 34.65 | 9.35 | 30.09 | 8.19 | 36.35 | 38.51 | 23.02 |
| L2 | 27.67 | 18.76 | 34.75 | 10.16 | 35.4 | 7.53 | 34.79 | 41.95 | 22.56 |
| L3 | 28.16 | 18.52 | 36.21 | 10.85 | 40.44 | 8.32 | 31.23 | 44.51 | 22.39 |
| L4 | 28.19 | 18.61 | 35.85 | 11.64 | 40.43 | 8.36 | 32.33 | 47.12 | 22.47 |
| L5 | 28.5 | 18.28 | 35.28 | 10.16 | 45.24 | 9.32 | 32.49 | 55.08 | 21.93 |

A: the transverse diameter of the vertebral body; B: the longitudinal diameter of the vertebral body; C: vertebral height; D: pedicle height; E: pedicle angle; F: length of the midline of pedicle; H: distance between right and left facet joints; I: the distance from the middle of the transverse process to the lower endplate.

Supplementary Table 2. Screw position measurements

|  | d (mm) | d1 (mm) | θ (°) | θ1 (°) | d' (mm) | d1' (mm) | θ' (°) | θ1' (°) |
| --- | --- | --- | --- | --- | --- | --- | --- | --- |
| Auto planning group |  |  |  |  |  |  |  |  |
| 1 | 20.4 | 20.55 | 62.95 | 64.97 | 21.12 | 20.66 | 18.62 | 18.61 |
| 2 | 21.36 | 21.11 | 53.92 | 59.29 | 18.87 | 19.23 | 20.18 | 20.06 |
| 3 | 19.18 | 18.86 | 52.58 | 53.89 | 27.98 | 28.06 | 17.31 | 16.94 |
| 4 | 19.26 | 19.45 | 61.97 | 64.62 | 22.04 | 25.25 | 16.67 | 16.25 |
| 5 | 14.73 | 21.20 | 64.32 | 66.65 | 22.21 | 22.35 | 18.23 | 18.32 |
| 6 | 20.84 | 21.35 | 57.65 | 61.22 | 27.00 | 20.05 | 21.19 | 21.57 |
| 7 | 18.67 | 18.48 | 60.04 | 62.94 | 20.67 | 20.75 | 17.98 | 18.43 |
| 8 | 19.02 | 15.15 | 64.61 | 68.12 | 19.02 | 18.87 | 21.04 | 20.46 |
| 9 | 21.58 | 21.01 | 63.52 | 65.73 | 27.29 | 27.90 | 22.32 | 22.26 |
| 10 | 19.70 | 19.71 | 62.73 | 65.56 | 21.96 | 21.96 | 18.32 | 19.29 |
| Manual planning group |  |  |  |  |  |  |  |  |
| 1 | 21.67 | 21.08 | 59.62 | 62.47 | 20.44 | 19.67 | 2.20 | 2.76 |
| 2 | 22.85 | 23.55 | 48.21 | 51.19 | 16.75 | 16.83 | 4.46 | 4.24 |
| 3 | 20.59 | 20.92 | 47.91 | 49.80 | 25.74 | 26.18 | 4.00 | 3.07 |
| 4 | 20.67 | 20.97 | 55.76 | 56.67 | 20.15 | 20.60 | 0.17 | 0.20 |
| 5 | 15.23 | 19.04 | 59.67 | 61.20 | 20.43 | 26.99 | 4.93 | 4.54 |
| 6 | 21.11 | 21.07 | 52.64 | 55.68 | 24.71 | 22.39 | 2.82 | 1.19 |
| 7 | 19.92 | 20.18 | 56.72 | 59.19 | 19.54 | 19.69 | 4.97 | 4.56 |
| 8 | 20.51 | 15.22 | 60.42 | 62.68 | 17.29 | 16.76 | 1.13 | 0.46 |
| 9 | 23.48 | 23.47 | 58.67 | 61.96 | 24.54 | 24.64 | 4.27 | 3.49 |
| 10 | 20.97 | 21.33 | 58.20 | 58.46 | 20.86 | 20.95 | 3.31 | 2.50 |

Supplementary Table 3. CT value measurements

|  | Control group | | Decalcification group | | | |
| --- | --- | --- | --- | --- | --- | --- |
|  |  |  | Before decalcification | | After decalcification | |
|  | CT value（Hu） | T' value | CT value（Hu） | T' value | CT value（Hu） | T' value |
| 1 | 2780.21 | 0.47 | 2755.94 | -0.46 | 2693.69 | -2.84 |
| 2 | 2792.36 | 0.94 | 2750.34 | -0.67 | 2677.88 | -3.44 |
| 3 | 2792.27 | 0.93 | 2773.86 | 0.23 | 2688.95 | -3.02 |
| 4 | 2749.31 | -0.71 | 2744.24 | -0.9 | 2685.61 | -3.15 |
| 5 | 2728.81 | -1.49 | 2811.37 | 1.66 | 2697.87 | -2.68 |
| Mean ± SD | 2768.59 ± 28.35 | 0.03 ± 1.08 | 2767.15 ± 27.08 | -0.03 ± 1.03 | 2688.8 ± 7.67 | -3.03 ± 0.29 |
